# Therapeutic effect of berberine on TDP-43-related pathogenesis in FTLD and ALS

**DOI:** 10.1186/s12929-016-0290-z

**Published:** 2016-10-21

**Authors:** Cheng-Fu Chang, Yi-Chao Lee, Kuen-Haur Lee, Hui-Ching Lin, Chia-Ling Chen, Che-Kun James Shen, Chi-Chen Huang

**Affiliations:** 1 Department of Neurosurgery, Taipei Medical University Hospital, Taipei, Taiwan; 2 Graduate Institute of Neural Regenerative Medicine, College of Medical Science and Technology, Taipei Medical University, Taipei, Taiwan; 3 Graduate Institute of Cancer Biology and Drug Discovery, College of Medical Science and Technology, Taipei Medical University, Taipei, Taiwan; 4 Institute and Department of Physiology, School of Medicine, National Yang-Ming University, Taipei, Taiwan; 5 Translational Research Center, Taipei Medical University, Taipei, Taiwan; 6 Institute of Molecular Biology, Academia Sinica, Nankang, Taipei Taiwan

**Keywords:** TDP-43, Berberine, mTOR, Autophagy, LC3-I/II

## Abstract

**Background:**

In the central nervous system regions of the sporadic and familial FTLD and ALS patients, TDP-43 has been identified as the major component of UBIs inclusions which is abnormally hyperphosphorylated, ubiquitinated, and cleaved into C-terminal fragments to form detergent-insoluble aggregates. So far, the effective drugs for FTLD and ALS neurodegenerative diseases are yet to be developed. Autophagy has been demonstrated as the major metabolism route of the pathological TDP-43 inclusions, hence activation of autophagy is a potential therapeutic strategy for TDP-43 pathogenesis in FTLD and ALS. Berberine, a traditional herbal medicine, is an inhibitor of mTOR signal and an activator for autophagy. Berberine has been implicated in several kinds of diseases, including the neuronal-related pathogenesis, such as Parkinson’s, Huntington’s and Alzheimer’s diseases. However, the therapeutic effect of berberine on FTLD or ALS pathology has never been investigated.

**Results:**

Here we studied the molecular mechanism of berberine in cell culture model with TDP-43 proteinopathies, and found that berberine is able to reverse the processing of insoluble TDP-43 aggregates formation through deregulation of mTOR/p70S6K signal and activation of autophagic degradation pathway. And inhibition of autophagy by specific autophagosome inhibitor, 3-MA, reverses the effect of berberine on reducing the accumulation of insoluble TDP-43 and aggregates formation. These results gave us the notion that inhibition of autophagy by 3-MA reverses the effect of berberine on TDP-43 pathogenesis, and activation of mTOR-regulated autophagy plays an important role in berberine-mediated therapeutic effect on TDP-43 proteinopathies.

**Conclusion:**

We supported an important notion that the traditional herb berberine is a potential alternative therapy for TDP-43-related neuropathology. Here we demonstrated that berberine is able to reverse the processing of insoluble TDP-43 aggregates formation through deregulation of mTOR/p70S6K signal and activation of autophagic degradation pathway. mTOR-autophagy signals plays an important role in berberine-mediated autophagic clearance of TDP-43 aggregates. Exploring the detailed mechanism of berberine on TDP-43 proteinopathy provides a better understanding for the therapeutic development in FTLD and ALS.

## Background

Deposition of the pathological signature proteins in the brain is the hallmark in various kinds of neurodegenerative diseases. The pathological signature in numerous neurodegenerative diseases are characterized by the accumulation of intracellular or extracellular protein aggregates composed of amyloid fibrils [1], such as senile plaques and neurofibrillary tangle composed of β-amyloid, microtubule associated protein tau in Alzheimer’s disease (AD), and Lewy bodies composed of α-synuclein in Parkinson’s disease (PD). Recently, the 43 kDa nuclear protein TAR DNA-binding protein (TDP-43) has been identified as a major component of UBIs aggregated proteins in sporadic and familial frontotemporal lobar degeneration with ubiquitinated inclusions (FTLD-U) as well as sporadic amyotrophic lateral sclerosis (ALS) [2, 3]. TDP-43 is a 414 amino acid nuclear protein encoded by the *TARDBP* gene on chromosome 1p36.2 and plays important roles in gene regulation at RNA transcription levels involving in transcriptional repression and alternative splicing [4, 5]. TDP-43 is highly conserved in various species including mammals, flies and caenorhabditis elegans [4, 6] and ubiquitously expresses in all tissues including brain [4, 7]. In about 95 % of ALS and 50 % of FTD cases, the UBI(+)-inclusions are predominately comprised of TDP-43 [8]. The pathological TAR DNA-binding protein is abnormally hyperphosphorylated, ubiquitinated, and cleaved into C-terminal fragments to form cytosolic aggregates in the central nervous system regions, including hippocampus, frontal cortex, temporal cortex, and spinal cord motor neuron [2]. Accumulation of TDP-43 within the Ub-positive insoluble aggregates implies that mis-regulation of the metabolism of TDP-43, including Ub-proteasome and autophagy pathways, plays a causative role in the pathogenesis. Indeed, numerous UPS and autophagy-related genes are mis-regulated in the frontal cortex samples of FTLD-TDP patients [9, 10]. Thus, identification of the potential drugs targeting on modulating the TDP-43 metabolism pathway might be a potential therapeutic strategy for FTLD-U and ALS patients with TDP-43 proteinopathies.

Induction of autophagy is a potential therapeutic target for accelerating the removal of aggregation-prone proteins [11]. Most of the misfolded/aggregated proteins are primarily degraded by autophagy-lysosomal pathway, which is negatively regulated by the activation of the kinase mammalian target of rapamycin (mTOR) pathway [12]. It has been well demonstrated that TDP-43 protein are degraded through both the ubiquitin-proteasome [13–15] and autophagy-lysosome metabolism pathways [16–19]. Nowadays, autophagy metabolism pathway has been shown to play an important role in TDP-43 degradation and aggregates removal. Depletion of functional multivesicular body (MVBs) required for the autophagy-lysosome degradation pathway results in the accumulation of the endogenous TDP-43 in the cytoplasm as ubiquitinated species [17]. Moreover, autophagy inhibition increases the accumulation of the C-terminal fragments of TDP-43 in cytosol, whereas inhibition of mTOR by rapamycin reduces the 25-kDa C-terminal fragments accumulation and restores TDP-43 localization in N2a and SH-SY5Y cells [16]. Autophagy activation by rapamycin rescues cytosolic TDP-43 mislocalization and truncated TDP-43 neurofilament instability in neurofibroblast cell lines [16]. The above results suggest that autophagy induction may be a valid therapeutic target for TDP-43 proteinopathies. The autophagy activator rapamycin is found to be a potential therapeutic drug in TDP-43-related pathogenesis [20–22], but its cytotoxicity resulted in the pathological failures have been observed in ALS model study [23]. Accordingly, searching for an alternative drug targeting on activating the autophagy pathway with less side effect would provide a better therapeutic effect for TDP-43 pathogenesis in FTLD and ALS.

The traditional herb medicine, berberine, is a potent autophagy activator through inhibition of the mTOR signal pathways [24, 25]. Recent studies have demonstrated that its high tolerance for orally-taken doses and the freely blood–brain-barrier permeability [26], make it an ideal alternative drug candidate for numerous neurodegenerative pathogenesis, including Alzheimer’s disease, Huntington’s disease and Parkinson’s diseases [27–31]. However, the therapeutic effect of berberine on TDP-43-related neuropathological diseases has never been discussed. Since berberine is a potent autophagy activator and activation of autophagy by mTOR inhibition would result in diminishing the TDP-43 accumulation and rescuing in memory/ motor function in rapamycin-treated cell culture and animal model [16, 22], we assumed that berberine can ameliorate TDP-43 proteinopathies. Here we found that berberine could promote the degradation rates and decrease the aggregates formation of truncated TDP-43 fragments through activating the autophagic function in cellular culture model, suggesting that berberine has potential neuroprotective effects on neurodegenerative diseases with TDP-43 proteinopathies.

## Methods

### Cell culture and DNA transfection

Neuro 2a (N2a) cells were cultured in Eagle’s minimum essential medium (Invitrogen, Carlsbad, CA, USA), supplemented with 10 % (vol/vol) fetal calf serum and penicillin/streptomycin (Invitrogen). DNA transfection of N2a cells was carried out with Lipofectamine 2000 (Invitrogen) according to the manufacturer’s instructions.

### Reagents, recombinant TDP-43 protein, and antibodies

Berberine is a kind gift from Dr. Kuan-Hau Lee. 3-MA (3-Methyladenine) was purchased from Sigma Aldrich (Saint Louis, MO, USA). **GFP-TDP-25**: The cDNA of human hTDP-43 (NM_007375.3) was cloned into the HindIII/KpnI restriction sites of pEGFP-C1 vector (Invitrogen). For generation of the truncated C-terminal hTDP-43 fragments (pGFPC1-hTDP-25) (amino acids 175–414), the DNA primers used for PCR amplification were as follows: forward primer 5’-CGGAAGCTTCGAATTCTAAGCAAAGCCAA-3’; and reverse primer 5’-CAGGTACCCTACATTCCCCAGCCAGA-3’. The PCR product was subcloned into the pEGFP-C1 plasmid (Invitogen) using restriction sites HindIII and KpnI to generate pGFP-C1-hTDP-25 (GFP-TDP-25). **Antibodies**: Rabbit polyclonal anti-GFP (Invitrogen). Rabbit polyclonal anti-LC3 (Sigma Aldrich; Cell Signaling, Danvers, MA, USA). Rabbit polyclonal anti-p-mTOR, anti-mTOR, anti-p-p70S6K and anti-p70S6K (Cell Signaling). Mouse monoclonal anti-α-tubulin (Sigma-Aldrich). Mouse monoclonal anti-GAPDH (Invitrogen).

### Preparation of cell extracts and immunoblotting

Cultured cell were washed twice in PBS and pelleted at 1,000 g for 5 min. To prepare the total cell extracts, the cell pellets were directly lysed in the urea buffer (Sigma-Aldrich). For preparation of RIPA-soluble and insoluble materials [32], the cell pellets were first lysed in the RIPA buffer (50 mM Tris–HCl, pH 8.0, 150 mM NaCl, 1 % NP-40, 0.5 % sodium deoxycholate, 0.1 % sodium dodecyl sulfate) freshly supplemented with complete EDTA-free protease inhibitor cocktail (Roche Applied Science, Laval, QC, CA) and phosphatase inhibitors (10 mM NaF and 1 mM Na_3_VO_4_) (Sigma-Aldrich). The protein concentrations of the lysates were determined by Bio-rad protein assay (Biorad, Marnes-la-Coquette, France) and then the lysates were centrifuged at 4 °C for 15 min at 12,000 rpm. The supernatants containing the RIPA soluble material were transferred into new tubes and boiled in SDS–PAGE sample buffer. The RIPA-insoluble pellets were washed twice in the lysis buffer, re-sonicated and re-centrifuged. The washed pellets were finally dissolved in the urea buffer, sonicated and supplemented with SDS–PAGE sample buffer without boiling. The soluble proteins and insoluble proteins were analyzed on SDS–PAGE gels and transferred onto polyvinylidene difluoride membranes (Immobilon-P, Millipore). After blocking for 1 h in the blocking buffer (5 % non-fat dry milk in Tris-buffered saline with 0.1 % Tween-20), the membranes were stained with the primary antibodies at 4 °C overnight and then the secondary antibodies at room temperature for 1 h. The bound antibodies were detected by using the chemiluminescence Western blotting detection reagent ECL (Amersham Pharmacia Biotech, Piscataway, NJ, USA).

### Immunofluorescence assay and aggregation analysis

N2a cells grown on glass coverslips were transfected with indicated plasmids. At 48 h post-transfection, the cells were treated with different inhibitors or drugs for different periods of time. The cells were fixed with 4 % ice-cold paraformaldehyde at 4 °C for 20 min and then permeabilized with PBS–0.5 % Triton X-100 for 7 min at RT. After blocking with 10 % donkey serum for 1 h at RT, the cells were incubated overnight at 4 °C with specific primary antibodies. After washing for three times with PBS, the cells were incubated at RT for 1.5 h with DAPI (1:500) (Invitrogen) plus Alexa Fluor 488-conjugated goat anti-rabbit or Alexa Fluor 488-conjugated goat anti-mouse IgG secondary antibody (1:500), or plus Alexa Fluora 546-conjugated goat anti-rabbit or Alexa Fluor 546-conjugated goat anti-mouse IgG secondary antibody (1:500) (Invitrogen). The images were examined on a LEICA DM6000B fluorescence microscope and quantified by MetaMorph software (Molecular Devices, Downingtown, PA). For quantitative analyses of aggregates, five representative fields per sample were taken and analyzed by MetaMorph software. GFP signal was gated to exclude non-transfected cells, and the images were then superimposed with corresponding DAPI images. The Metamorph was used to count the total number of transfected cells. For aggregate analyses, the GFP images were visually adjusted to determine a common threshold across all samples to eliminate diffuse or non-aggregated signals. The numbers of individual aggregates were calculated with the Integrated Morphometry Analysis of Metamorph. The percentages of aggregated cells were calculated as the following formula: total number of GFP-positive aggregated cells/total number of transfected cells. Statistical significance was analyzed with the student *t*-test.

## Results

### The effect of berberine on TDP-43 pathogenesis

**Fig. 1:**
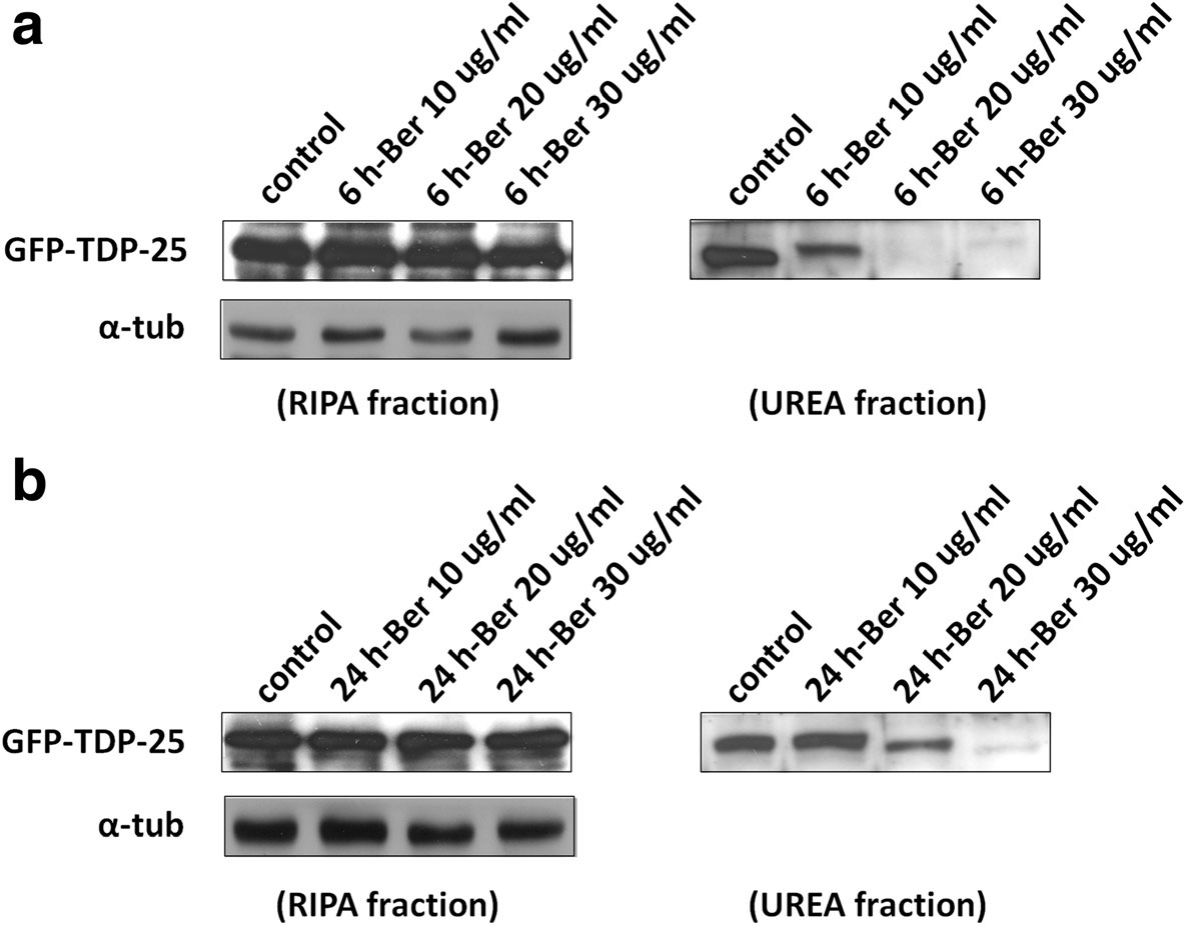
The effect of berberine on insoluble TDP-43 accumulation. **a** and **b** After 6 h (**a**) and 24 h (**b**) transfection, N2a cells were transfected with GFP-tagged aggregation-prone TDP-43 fragments (GFP-TDP-25) and treated with different dose of berberine for another 24 h. The soluble/insoluble TDP-43 were extracted by RIPA/urea fractionation and analyzed by Western blotting with anti-GFP antibody

**Fig. 2:**
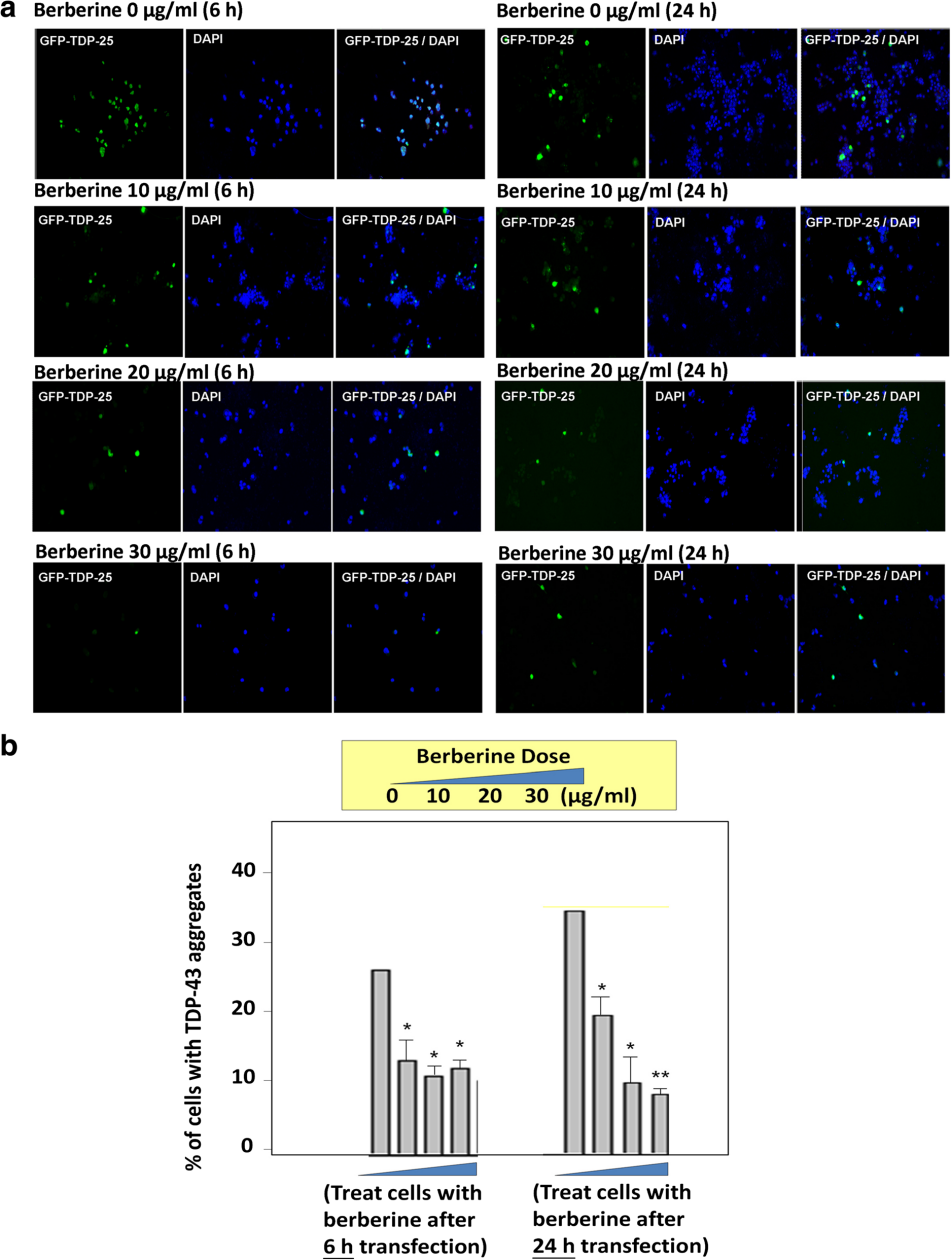
The effect of berberine on TDP-43 aggregates formation. **a** GFP-tagged aggregation-prone TDP-43 fragments (GFP-TDP-25) were transfected in N2a cells. After 6 h or 24 h transfection, N2a cells were treated with different dose of berberine for another 24 h. GFP-TDP-25-transfected N2a cells were then stained with anti-GFP antibody (green) in immunofluorescence assay. The nuclei were stained with DAPI (blue). **b** The histogram showing the percentage of cells with GFP-TDP-43 aggregates. Data are presented as mean ± s.e.m. of three independent experiments *p < 0.05; **p < 0.01 (Student’s *t*-test compared with the control)

First, we were interested in investigating the effect of the autophagy activator berberine on TDP-43 insoluble deposition formation. To observe whether berberine can prevent or dissolve TDP-43 aggregates, we overexpressed GFP-tagged aggregation-prone C-terminal TDP-43 fragments, GFP-TDP-25 [33, 34], and treated cells with different dose of berberine before or after the initiation of TDP-43 aggregates formation. Cells transfected with truncated C-terminal TDP-43 fragments (TDP-25) started to form insoluble cytosolic aggregates after 6 h transfection. Accumulation of truncated TDP-43(+) UBIs aggregates in cytosol in FTLD and ALS brains were identified as RIPA-insoluble species and most of the aggregates are dissolved in urea-soluble fractions in Western blot assay. To evaluate the neuroprotective effect of berberine on preventing insoluble TDP-43 formation, after 6 h transfection, the GFP-TDP-25-transfected cells were treated with various dose of berberine for another 24 h, and then the insoluble TDP-43 were extracted with RIPA and urea buffer to dissect the soluble and insoluble TDP-43 fractions and analyzed by Western blotting (sequential extraction method, please see Materials and Methods). Comparing to control group, the insoluble fraction of TDP-43 (urea fraction) was significantly lower in cells treated with berberine after 6 h transfection in a dose-dependent manner (Fig. 1a), suggesting that berberine can provide neuroprotective effect to prevent insoluble TDP-43 accumulation. To further validate the effect of berberine on inhibiting the formation of pathological TDP-43 inclusion, the percentage of TDP-43 aggregates in cells treated with berberine after 6 h transfection were measured and quantified by immunofluorescent assay. Comparing to control group, the aggregation percentage of TDP-43 was significantly lower in cells treated with berberine after 6 h transfection in a dose-dependent manner, especially in cells treated with 20 and 30 ug/ml of berberine (left panal in Fig. 2a and b), suggesting that berberine not only decreased the deposition of insoluble TDP-43 species, but also prevented the TDP-43 aggregate formation.

To further monitor the potential therapeutic role of berberine on dissolving the insoluble aggregated TDP-43, we treated cells with berberine in GFP-TDP-25-transfected N2a cells after TDP-43 aggregates formation. After 24 h transfection, the GFP-TDP-25-transfected cells were treated with different doses of berberine for another 24 h and the insoluble TDP-43 fractions were extracted by sequential extraction method and analyzed by Western blotting. Comparing to control group, the insoluble fraction of TDP-43 (urea fraction) showed a dose-dependent decrease in cells treated with berberine after 24 h transfection, (Fig. 1b), suggesting that berberine can also reverse the insoluble TDP-43 accumulation. To further validate the therapeutic effect of berberine on TDP-43 aggregates formation, the percentage of TDP-43 aggregates were measured and quantified by immunofluorescent assay in cells treated with berberine for 24 h after 24 h post transfection. Comparing to control group, the aggregation percentage of TDP-43 was significantly lower in cells treated with berberine, especially in cells treated with 20 and 30 ug/ml of berberine (right panal in Fig. 2a and b). These results demonstrated that berberine can both prevent and ameliorate the pathological TDP-43 formation.

### The mTOR-signaling pathway is involved in berberine-mediated autophagy activation and TDP-43 aggregates elimination

**Fig. 3:**
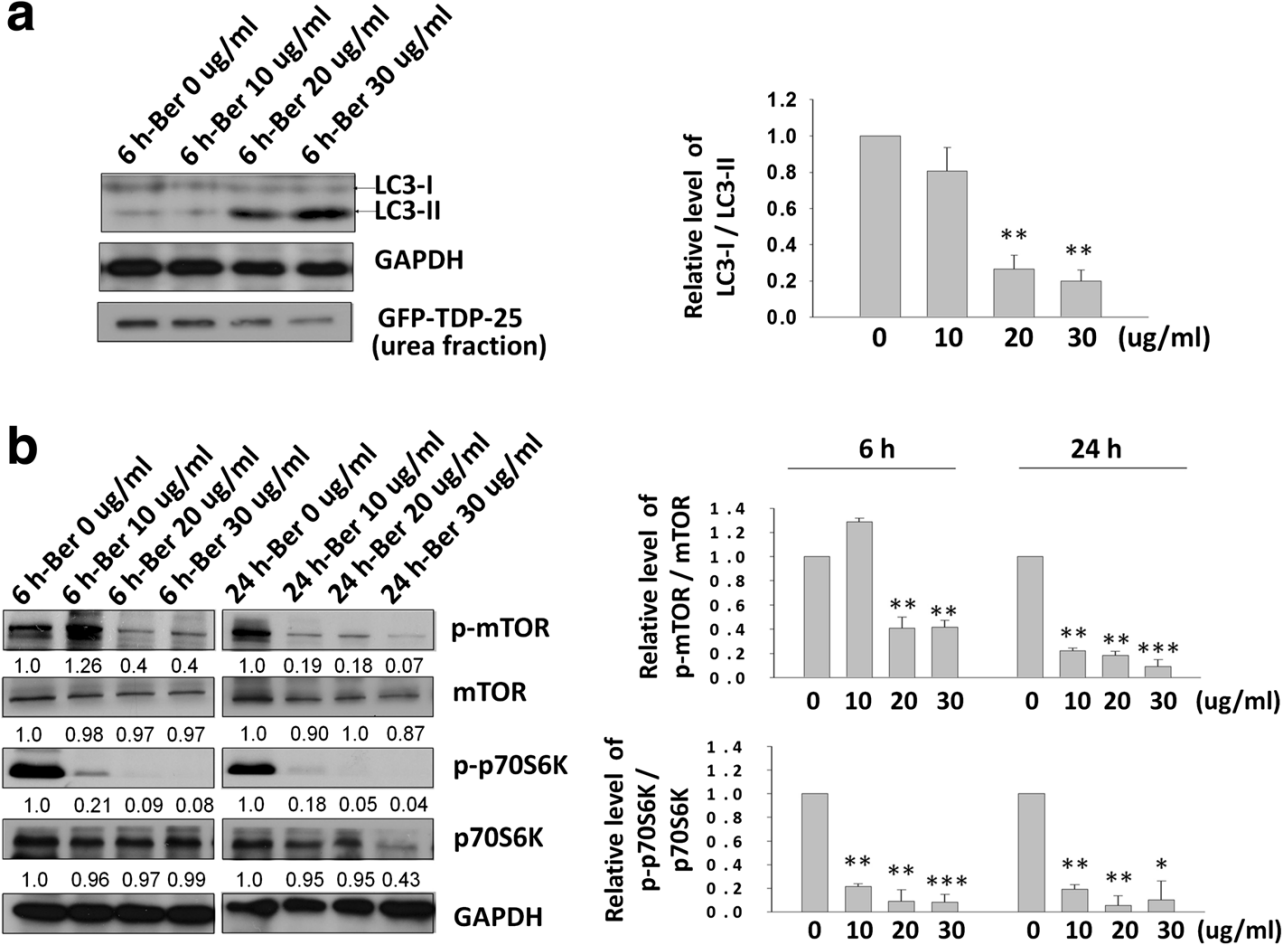
The mTOR/p70S6K-signaling pathway is involved in berberine-mediated autophagy activation and TDP-43 aggregates diminishing. **a** and **b** N2a cells were transfected with GFP-TDP-25 for 6 h or 24 h and then treated with berberine for another 24 h. Cell lysates were analyzed by Western blotting with anti-GFP and anti-LC3, anti-p-mTOR, anti-mTOR, anti-p-p70S6 and anti-p70S6 antibodies. Data are presented as mean ± s.e.m. of three independent experiments *p < 0.05; **p < 0.01; ***P < 0.005 (Student’s *t*-test compared with the control)

**Fig. 4:**
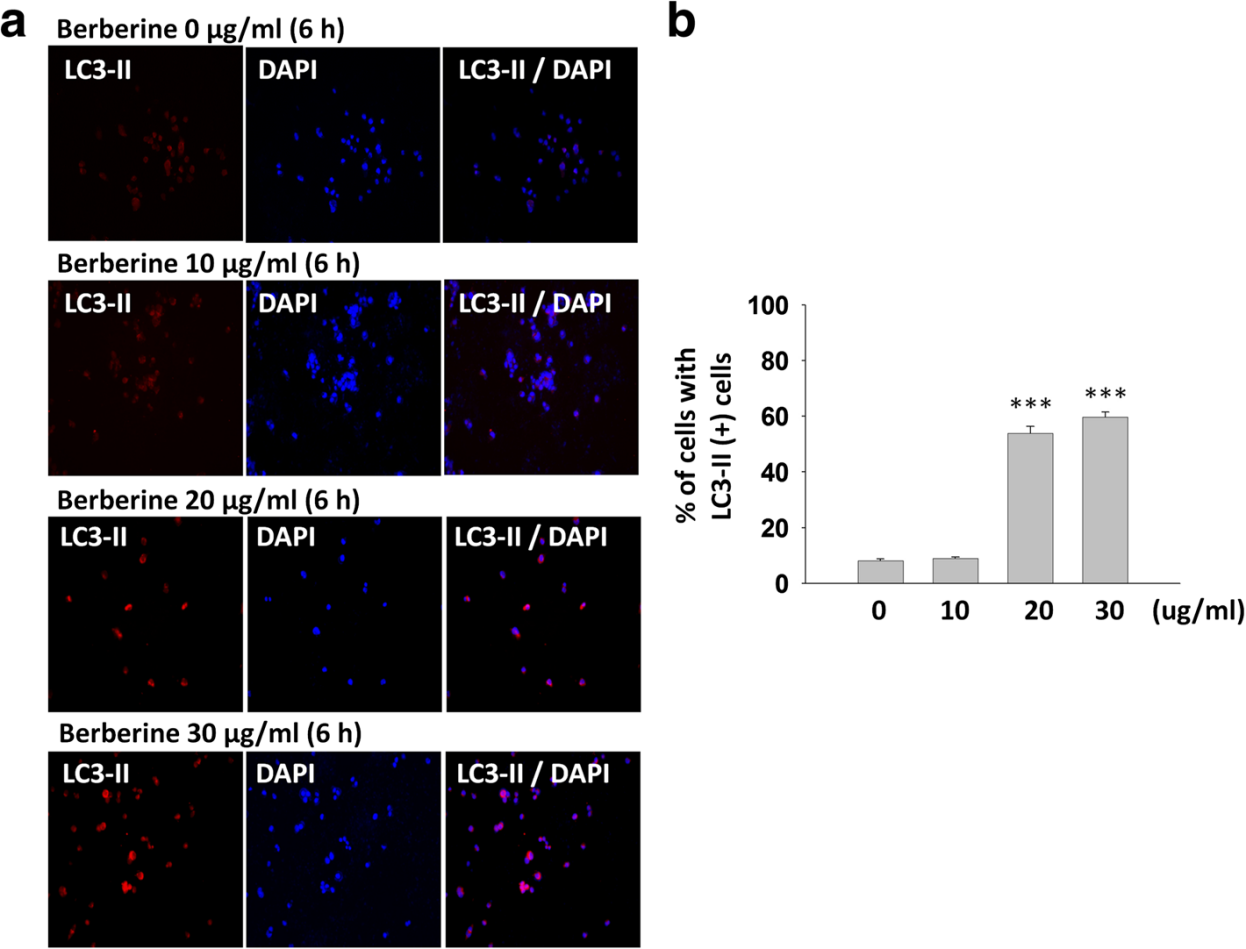
The autophagy activation marker LC3-II is significantly increased in berberine-treated cells. **a** N2a cells were transfected with GFP-TDP-25. After 6 h transfection, N2a cells were treated with different dose of berberine for another 24 h, and then stained with anti-LC3-II (red) in immunofluorescence assay. The nuclei were stained with DAPI (blue). **b** Data are presented as mean ± s.e.m. of three independent experiments ***P < 0.005 (Student’s *t*-test compared with the control)

It has been demonstrated that berberine is a potent autophagy activator through inhibiting mTOR signaling pathways [24, 25]. Autophagy is the major catabolic process to remove bulk cytoplasmic contents, abnormal protein aggregates, and excess or damaged organelles. The effect of berberine on reducing TDP-43 aggregates led us to hypothesize that berberine affected the aggregated TDP-43 processing through mTOR inhibition and autophagy activation. To investigate whether mTOR-regulated autophagy process is involved in berberine-mediated clearance of pathogenic TDP-43, we examined the levels of autophagic and mTOR activity markers, LC3-II protein, by Western blotting. Reversion of LC3-I into LC3-II is an indicator as the formation of autophagosome in autophagy system [35]. After 6 h post transfection, different dose of berberine were added into GFP-TDP-25-transfected N2a cells for another 24 h. The ratio of LC3-I/LC3-II were analyzed by Western blotting and compared by densitometry. A lower ratio of LC3-I/LC3-II indicates more conversion of LC3-I to LC3-II during synthesis of the autophagosome and activation of autophagy [35]. In GFP-TDP-25-transfected cells treated with different dose of berberine, the insoluble TDP-43 species and protein ratio of LC3-I/LC3-II in berberine-treated cells were both decreased in a dose-dependent manners, comparing to that of control group (Fig 3a), suggesting that berberine-mediated autophagosome formation and autophagy activation is involved in diminishing insoluble TDP-43 formation. It has been demonstrated that activation of mTOR by phosphorylation would further phosphorylate and activate p70S6K [36, 37]. Accordingly, we further detected the phosphorylation level of both mTOR and p70S6K in berberine-treated cells. After 6 h or 24 h post transfection, different dose of berberine were added into GFP-TDP-25-transfected N2a cells for another 24 h. Either in 6 h or 24 h post transfected-cells, compared with control group, both of the phosphorylated mTOR and p70S6K in cells treated with berberine is significantly decreased in which their protein levels were unchanged (Fig. 3b). Moreover, consistent with the observation in Fig. 3a, we found that the autophagosome marker LC3-II represented in autolysosomes structure was significantly enhanced in berberine-treated cells with higher dose compared with control group (Fig. 4a and b). The above results demonstrated that treatment of berberine would inhibit the mTOR signaling cascades and activate the autophagy pathway to decrease the pathological TDP-43 formation.

### Inhibition of autophagy reverses the effect of berberine on TDP-43 pathogenesis

**Fig. 5:**
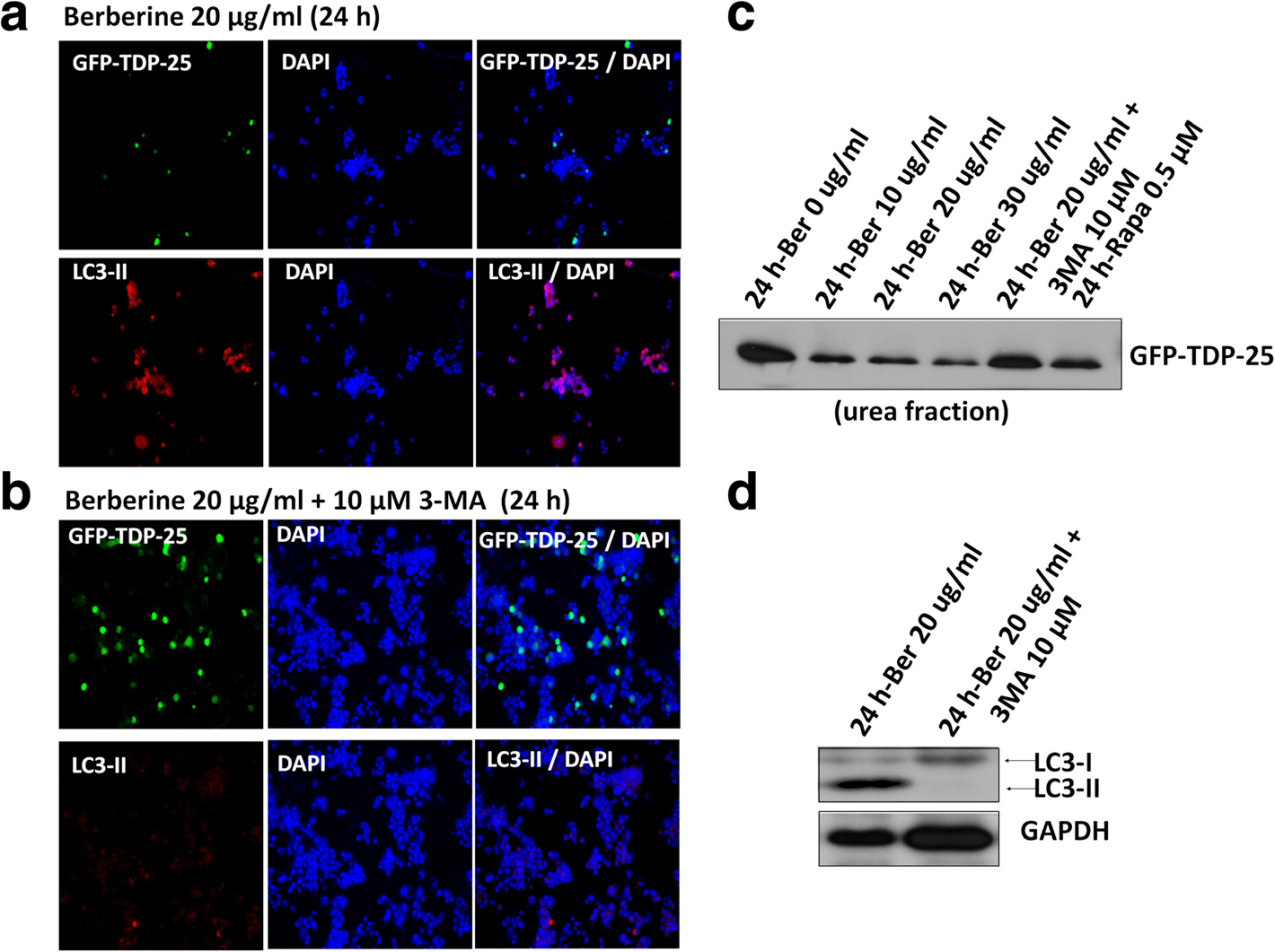
Inhibition of autophagy reverses the effect of berberine on TDP-43 aggregates elimination. **a** and **b** After 24 h transfection, N2a cells transfected with GFP-TDP-25 were treated with 20 μg/ml berberine and autophagy inhibitor 10 μM 3-MA for another 24 h, and stained with anti-GFP (green) and anti-LC3-II (red). **c** and **d** The soluble/insoluble TDP-43 were extracted by RIPA/urea fractionation and analyzed by Western blotting with anti-GFP and anti-LC3 antibodies

**Fig. 6:**
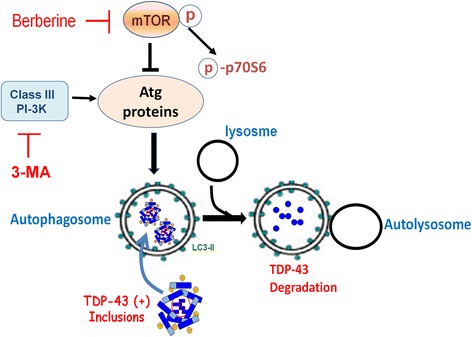
Schematic diagram of the mechanism of berberine on reversing the aggregated TDP-43 processing through mTOR inhibition and autophagy activation in cells with TDP-43 pathogenesis

To further confirm that the effect of berberine on decreasing TDP-43 aggregates is mediated by autophagy activation, we inhibited autophagy by specific autophagosome inhibitor, 3-MA (3-Methyladenine). 3-MA inhibits autophagy by blocking autophagosome formation via inhibiting the mTOR key regulator, class III Phosphatidylinositol 3-kinases (PI-3 K), and is broadly used to study autophagy-related mechanism under various kinds of cellular condition [38]. We examined the TDP-43 aggregates by immunofluorescent and Western blot assays in GFP-TDP-25-transfected N2a cells treated with berberine only or combined with 3-MA. The therapeutic effect between 20 and 30 ug/ml of berberine in TDP-43-aggregated cells were comparable (Fig. 1 and 2), yet cells treated with 30 ug/ml of berberine showed minor cytotoxicity with relatively lower total cell numbers (Fig. 2). Accordingly, 20 μg/ml berberine was used to study the inhibition effect of 3-MA on berberine-mediated decrease of TDP-43 aggregates. After 24 h post transfection, GFP-TDP-25-transfected N2a cells were added with 20 μg/ml berberine or 10 μM 3-MA for another 24 h, and the TDP-43 aggregates were identified under microscopy. In berberine-treated cells, GFP-TDP-25 was obscure yet the autophagosome marker LC3-II were significantly increased (Fig. 5a). However, in berberine and 3-MA co-treated cells, the GFP-TDP-25 was prominently enhanced but the signal of LC3-II was obviously reduced (Fig. 5b and d). Moreover, 3-MA also reversed the effect of berberine on reducing the accumulation of insoluble fraction of TDP-43 (urea fraction) in Western blot assay (Fig. 5c). And the levels of insoluble TDP-43 protein in berberine-treated cells was even lower than that of cells in 0.5 μM rapamycine-treated cells (Fig. 5c), suggesting that the effect of berberine is better than rapamycin on diminishing insoluble TDP-43 accumulation. These results give us the notion that inhibition of autophagy by 3-MA reverses the effect of berberine on TDP-43 pathogenesis, and activation of mTOR-regulated autophagy plays an important role in berberine-mediated therapeutic effect on TDP-43 proteinopathies (Fig. 6).

## Discussion

Most of the short-lived proteins are degraded by UPS through the 26S proteasome [39], whereas the autophagy-lysosomal pathway primarily catabolizes unnecessary organelles, long-lived proteins, and misfolded/aggregated proteins [12]. To date, several studies have reported that full-length TDP-43 protein and its truncated 25 kDa and 35 kDa fragments are degraded through both the ubiquitin-proteasome [13–15] and autophagy-lysosome metabolism pathways [16–18]. TDP-43 is not only a substrate for autophagy, but also as a maintenance factor of the autophagy system by stabilizing the mRNA of autophagy-related genes, ATG7 [40]. Autophagy is negatively regulated by mTOR [11]. Induction of autophagy would accelerate the removal of aggregation-prone proteins and provides a neuroprotective effect in several neurodegenerative disease models, including Huntington disease [41], Alzheimer’s disease [42], Parkinson disease [43]. The autophagy activator, rapamycin, which is an antifungal and immunosuppressant compound that targeting in inhibition of the mTOR signal, has been identified its potential therapeutic effect on numerous neurodegenerative diseases including TDP-43-related pathogenesis [20–22]. However, the cytotoxicity of rapamycin leading to the pathological failures have also been reported in Alzheimer’s disease [44, 45] and ALS model study [23], which restricted its clinical therapeutic application. Thus, an alternative new drug targeting on activating the autophagy pathway with less side effect would provide better therapeutic effect on these neurodegenerative diseases including the TDP-43 pathogenesis.

Recent studies have demonstrated that the traditional herb medicine, berberine, is an isoquinoline alkaloid found in a number of important medicinal plant species and is a potent autophagy activator through inhibition of the mTOR signal pathways [25, 30]. Berberine is a long-term used herb medicine applied in broad clinical diseases and is even used as dietary nutritional supplement or orally-taken drug since its low side effect and cellular toxicity. Several studies have reported that the natural compound berberine, with either the extract or pure berberine, is safely administered to humans, and is used in various kinds of diseases, such as antibacterial, anti-hypertensive, anti-inflammatory, anti-diabetic, anti-hyperlipidemic and anti-cancer effects [46, 47]. Berberine can be safely used to treat a number of diseases, and even partly replace the commercial drugs, which could lead to a reduction in toxicity and side effects [48]. Preliminary results suggest the initiation of clinical trials in patients with neuronal related diseases, such as depression, bipolar affective disorder, schizophrenia, or related diseases in which cognitive capabilities are affected [49, 50]. The high tolerance for orally-taken doses (LD50 > 5 g/kg) and the freely blood–brain-barrier permeability of berberine [26] make it an ideal drug candidate for chronic neurological disorders. Recently, several studies have found that berberine is a potential neuroprotector for several neurodegenerative pathogenesis, including Parkinson’s disease, Huntington’s diseases and Alzheimer’s disease (AD) through ameliorating the β-amyloids deposition, or enhancing the degradation of mutant huntingtin by autophagic pathway [27–31]. Here we also demonstrated that berberine promotes the metabolism rates and decrease the aggregates formation of truncated TDP-43 fragments through activating the autophagic function in cellular culture model, implying its potential neuroprotective effects on neurodegenerative diseases with TDP-43 proteinopathies.

Berberine also exhibits multifaceted pharmacological effects on neuroprotection or againsting neurodegeneration through shared signaling pathways. In both Alzheimer’s and Parkinson’s patients, monoamine oxidase B (MAO-B) activity is elevated and MAO-B-mediated dopamine metabolism pathway leading to H_2_O_2_ generation would cause oxidative stress and reactive oxygen species (ROS)-mediated damage [51]. Berberine-counteracted MAO-B and ROS activity has positive effects on againsting pro-apoptotic damage in numerous neurodegenerative diseases [29, 52, 53]. Additionally, berberine also attenuates hydrogen peroxide-induced neurotoxicity through up-regulation of nuclear factor erythroid 2 (Nrf2), glycagon-like protein 1 (GLP-1), as well as phosphorylation of Protein kinase B (AKT) and cyclic adenosine monophosphate response element- binding protein (CREB) [54–58]. GLP-1 plays a role in preservation of dopaminergic neurons and phosphorylated CREB is essential for neurons to survive damage [59–62]. Furthermore, AChE would induce neuronal apoptotic and berberine-mediated inhibition of AChE has broadly effect on a great number of neurodegenerative diseases [63]. Berberine exhibits broad neuroprotective properties through various known signaling pathways. As for the adverse effects of berberine, limited evidence has been reported. It has been reported that a dose of 5–15 mg/kg berberine would decrease the number of dopaminergic neurons in the substantia nigra and the striatum, suggesting its toxic effect on disturbing the motors and cognitive functions [64]. Furthermore, berberine would inhibit the dopamine synthesis and enhance neurotoxicity of 6- hydroxydopamine in cell culture system leading to neurotoxic at the dose of 10–30 mM [65]. Recently, berberine was found to cause mitochondria-dependent toxicity and sensitize neurons to the glutamate excitotoxicity in cultured primary neurons [66]. To better understand the toxicity of the berberine, exploring the potential side effects of berberine is indispensible.

Berberine has been identified as a potent autophagy activator through inhibiting mTOR signaling pathways [24, 25]. Autophagy is a catabolic process that results in the autophagosomic-lysosomal degradation of bulk cytoplasmic contents, abnormal protein aggregates, and excess or damaged organelles. Autophagy is inhibited by the mTOR signaling and generally activated by conditions of nutrient deprivation, oxidation, infection and tumor suppression [67]. Activation of mTOR by Akt or MAPK signaling would further phosphorylate and activate its downstream serine/threonine kinase p70S6K, and also suppress the initiation of a cascade of autophagy related genes (Atg) expression and autophagy activation [36, 37]. Previous studies have demonstrated that berberine possesses multiple activities in Alzheimer's disease therapy, including antioxidant activity, AChE and BChE inhibitory activity, MAO inhibitory activity, and reducing Aβ level and lowering cholesterol [58]. Berberine can be administered orally and pass through the blood–brain barrier [26, 68], suggesting its potential effect for FTLD and ALS. Here we found that berberine reverses the aggregated TDP-43 processing through inhibition of mTOR/p70S6K signal and autophagy activation in FTLD and ALS pathogenesis. In addition, the ratios of LC3-I/LC3-II in berberine-treated N2a cells were significantly decreased (Fig 3a), indicating that berberine enhances autophagosome formation and further triggers the following autolysosome activation. When we inhibited autophagy by specific PI-3 K inhibitor, 3-MA, which specifically inhibits the formation of autophagosome during autophagy process, a reverse effect of berberine on autophagy activation and TDP-43 aggregates elimination were observed (Fig. 5). These results further confirming that activation of mTOR-regulated autophagy plays an important role in berberine-mediated therapeutic effect on TDP-43 proteinopathies (Fig. 6). We explored the detailed molecular mechanism of berberine on TDP-43 metabolism and insoluble TDP-43 deposition, and provided a better alternative therapeutic strategy for FTLD and ALS diseases.

## Conclusions

We supported an important notion that the traditional herb berberine is a potential alternative therapy for TDP-43-related neuropathology, Here we demonstrated that berberine is able to reverse the processing of insoluble TDP-43 aggregates formation through deregulation of mTOR/p70S6K signal and activation of autophagic degradation pathway. mTOR-autophagy signals plays an important role in berberine-mediated autophagic clearance of TDP-43 aggregates. Exploring the detailed mechanism of berberine on TDP-43 proteinopathy provides a better understanding for the therapeutic development in FTLD and ALS.

## Abbreviations

3-MA: 3-Methyladenine
AD: Alzheimer’s disease
ALS: Amyotrophic lateral sclerosis
FTLD-U: Frontotemporal lobar degeneration
mTOR: Mammalian target of rapamycin
MVBs: Multivesicular body
N2a: Neuro 2a
PD: Lewy bodies composed of α-synuclein in Parkinson’s disease
PI-3 K: Class III Phosphatidylinositol 3-kinases
TDP-43: 43 kDa nuclear protein TAR DNA-binding protein
